# Cannabis use and the risk of tuberculosis: a systematic review

**DOI:** 10.1186/s12889-019-7127-0

**Published:** 2019-07-27

**Authors:** Clare E. French, Caroline M. Coope, Luke A. McGuinness, Charles R. Beck, Sophie Newitt, Lauren Ahyow, Matt Hickman, Isabel Oliver

**Affiliations:** 1 Population Health Sciences, Bristol Medical School, University of Bristol, Bristol, BS8 2PS UK; 2 National Institute for Health Research Health Protection Unit in Evaluation of Interventions, Bristol Medical School, University of Bristol, Bristol, BS8 2PS UK; 3 Field Service South West, National Infection Service, Public Health England, Bristol, BS1 6EH UK; 4 Field Service East Midlands, National Infection Service, Public Health England, Nottingham, NG2 4LA UK; 5 Public Health England East Midlands, Public Health England, Nottingham, NG2 4LA UK

**Keywords:** Tuberculosis, Cannabis, Systematic review, Evidence synthesis

## Abstract

**Background:**

Cannabis has been identified as a possible risk factor in some tuberculosis (TB) outbreaks. As the most widely used (largely) illegal substance in Western countries this may be an important public health concern. We aim to systematically review the evidence on the association between cannabis use and TB (latent infection and active disease) to inform ongoing and future TB prevention and control strategies.

**Methods:**

We conducted a systematic review. We searched Ovid Medline, Embase and PsycInfo, together with the World Health Organization website and Google Scholar, for all years to January 2018. Reference lists and conference abstracts were hand-searched, a forward citation search was conducted on the Web of Science, and experts were contacted. Two authors independently screened studies for inclusion, extracted data and assessed risk of bias using an adapted version of ROBINS-I (“Risk of Bias in Non-randomised Studies – of Interventions”). Data were narratively synthesised.

**Results:**

Of 377 records identified, 11 studies were eligible. Study designs were heterogeneous. Six studies utilised a relevant comparator group. Four of these investigated the association between cannabis use and latent TB infection; all provided some evidence of an association, although only two of these had adjusted for confounders. The remaining two comparator studies investigated the association between cannabis use and active TB disease; neither found evidence of an association after adjusting for confounding. All six studies were at “Serious” risk of bias. The five studies which did not utilise a relevant comparator group were all indicative of TB outbreaks occurring among cannabis users, but the quality of the evidence was very weak.

**Conclusions:**

Evidence for an association between cannabis use and TB acquisition is weak. The topic warrants further robust primary research including the collection of consistent and accurate exposure information, including cannabis use practices, dose and frequency, and adjustment for confounders.

**Electronic supplementary material:**

The online version of this article (10.1186/s12889-019-7127-0) contains supplementary material, which is available to authorized users.

## Background

It is estimated that 1.7 billion people are infected with *Mycobacterium tuberculosis* (TB) globally of which 5 to 15% will develop active TB disease, depending on co-existing risk factors such as under-nutrition, diabetes, HIV (human immunodeficiency virus) infection, smoking and alcohol consumption [1]. In high-income countries TB has most commonly occurred in marginalised populations such as migrants, the homeless, people who inject drugs and prisoners [2–4]. With the introduction of whole genome sequencing in TB outbreak investigation the accurate linkage of cases, even prior to epidemiological linkage [5], has facilitated the identification of previously unrecognised risk factors for transmission. These novel risk factors, such as cannabis use, may be important to inform outbreak investigations and control efforts and need to be better understood.

Cannabis is estimated to be the most widely used (largely) illegal substance in Western countries including Europe [6], North America [7] and Australia [8]. Frequent cannabis use has been reported among cases in previous TB outbreaks [9, 10], and recently in an outbreak of pulmonary TB in the United Kingdom (UK) in which 26% of those with latent TB infection (LTBI) and 67% with TB disease reported cannabis smoking (Lauren Ahyow, Public Health England – personal communication). Given the prevalence of cannabis use, and the increasing potency available [6], there is a pressing need to understand whether there is an association between cannabis use and TB - a potentially significant public health concern.

The method used to inhale cannabis could be important in TB transmission and links have been found with sharing a cannabis water pipe or ‘bong’ [11] and ‘shotgunning’ (the practice of inhaling smoke and then exhaling it into another individual’s mouth) [12]. These behaviours may offer an environment for the efficient transmission of TB, such as prolonged exposure and close proximity to a case. Evidence supports the biological plausibility of an association as heavy cannabis smoking has been associated with chronic bronchitis symptoms and large airway inflammation which may lower the lungs natural defences against infection [13]. Additionally, there is evidence that tobacco smoking, often adjunct to cannabis use, is associated with TB infection and disease [14] and may increase the risk of disease by as much as 2.5 times [15].

To our knowledge, evidence on the possible association between cannabis use and TB infection and disease has not been systematically reviewed. We aim to address this gap to inform ongoing and future TB prevention and control strategies.

## Methods

This review is reported in accordance with the Preferred Reporting Items for Systematic Reviews and Meta-Analyses (PRISMA) [16]. The review protocol can be obtained by request from the corresponding author.

### Eligibility criteria

All types of primary epidemiological studies (e.g. descriptive studies, outbreak reports, cohort studies, case-control studies) were eligible for inclusion. The review question was structured using a PECO framework as follows:- **P**opulation: adults aged ≥16 years; **E**xposure: cannabis use by any means; **C**omparator: any e.g. no reported cannabis use, no comparator; **O**utcome: active TB disease affecting any clinical site (pulmonary or extra-pulmonary) or latent infection e.g. assessed by Tuberculin Skin Testing [TST] (‘Mantoux’ test) or an interferon-gamma release assay. No restriction was placed on the type/availability of a specific comparator group in order that the complete body of evidence could be reviewed.

### Search strategy

Electronic searches were conducted in Ovid Medline, Embase and PsycInfo, from the inception of each database to 17 January 2018. Subject Headings (MeSH, EMTREE, APA) and free-text key words were used (see Additional file 1 for search terms). No restriction was placed on language or publication status. The World Health Organization website and Google Scholar were searched. We also checked abstract lists for the annual Union World Conference on Lung Health for the years 2015–2017. Reference lists of eligible studies were hand-searched, and a forward citation search was conducted in Web of Science to help identify follow-up studies or new research citing any of the study reports included in the review. Experts in the field were contacted to identify any further published or unpublished studies. Records were stored and managed in EndNote X9.

### Study selection

Two authors screened studies for eligibility and agreed the final list for inclusion. Reasons for exclusion of studies that reached the full-text screening stage were documented.

### Data extraction and synthesis

Data from eligible studies was double extracted by two reviewers (CEF and CMC) using a pre-defined form, and cross-checked. Key data items (as listed in Table 1) were extracted and narratively synthesized. Summary measures reported varied; we extracted odds ratios (e.g. comparing the odds of TB in those who reported using cannabis vs. those who did not) whenever documented. Studies which utilised a relevant comparator group are reported separately from those which did not. Given the substantial heterogeneity between studies it was not appropriate to perform a meta-analysis.

**Table 1:** Summary of studies included in the review

Author, year	Country	Study objective	Study type and description	Exposure - type of cannabis use (if reported)	TB case definition / method of diagnosis	Identification of contacts / method of diagnosis	Number of TB cases and number of contacts screened (as relevant)	Socio-demographics of TB cases	Key findings
Studies with a comparator group									
Morano, 2014 [21]	United States	To evaluate the efficacy of a mobile medical clinic screening program for detecting latent and active TB	Analysis of data routinely collected by the clinic, January 2003 to June 2011	Ever used cannabis. No further details provided	Latent TB was identified through TST. Those with a positive TST (≥10 mm for HIV-negative individuals and ≥ 5 mm for HIV-positive individuals) were assessed for active disease by screening for symptoms and chest X-ray. Those with symptoms or abnormal chest X-ray results underwent sputum culture	N/A	4650 people were screened of which 779 were identified as prevalent TB cases (newly and previously diagnosed)	Of all those with TB (newly or previously diagnosed), 62% were male, 48% foreign-born	Cannabis use was associated with incident (but not prevalent) TB infection in adjusted analyses - aOR: 1.57; 95% CI:1.05–2.37. Adjusted for foreign-born, unstable housing, Hispanic ethnicity, Black ethnicity, employed, age, hazardous drinking, crack cocaine use
Munckhof, 2003 [11]	Australia	To report a cluster of TB cases and investigate whether shared use of a cannabis water pipe was associated with TB transmission	Retrospective cohort study / outbreak investigation	Smoking a cannabis water pipe (‘bong’)	Active TB cases were identified via contact screening and self-referral and diagnosis confirmed with clinical examination and positive sputum culture (all isolates were identical on typing)	All contacts were screened with an initial TST and chest radiograph. If the initial TST was not significant, a second TST was performed 3 months after the last contact with the relevant index case	5 pulmonary TB cases (all were culture positive, 4 of whom were sputum AFB smear positive), 149 identified contacts	All cases were young (median age 20 years), Australian-born, HIV-negative, Caucasian males	Of 45 contacts who had shared a cannabis bong with a case, 29 (64%) had a significant TST reaction. There was some (weak) evidence that sharing a cannabis water pipe with a pulmonary TB case was associated with an increased risk of acquiring TB infection (univariable association *p* = 0.05 [odds ratio not presented], adjusted OR: 2.22, 95% CI 0.96–5.17). The model adjusted for gender of contact, whether shares a house with case, and previous BCG vaccination. In this cluster, cannabis smoking was frequently performed in closed rooms or cars
Thu, 2013 [22]	Australia	To report on 3 cases of cavitating disease, which seemed to be associated with smoking cannabis using a makeshift pipe or bong	Case series plus retrospective cohort of contacts - reports on 3 cases of active TB and results of the screening of their contacts	Smoking a cannabis water pipe ‘bong’	The 3 index cases all had cavitating pulmonary TB and were AFB sputum smear positive	Contacts were screened using TST	3 TB cases who had a total of 111 identified contacts (34 were positive for latent TB, one of whom developed active disease)	All index cases were young Australian-born, non-indigenous adults	All 3 index cases regularly smoked cannabis through a bong. The unadjusted odds of having TB infection (positive TST) in contacts who shared a bong with an active TB case vs. those who did not report doing so was 6.5, 95% CI: 1.4–30.4, p = 0.016
Oeltmann, 2006 [9]	United States	To describe a TB outbreak fuelled by illicit drug use	Retrospective cohort study / outbreak investigation. Isolates from all culture positive TB patients in Seattle and King County, Washington, 2003–2004 were genotyped. Patients who had an isolate that matched the outbreak strain or who had a social link to an already included patient were included in the study	‘Hotboxing’ (all cases reported frequent hotboxing)	TB cases were those that were culture-confirmed	Contacts were defined as friends or others. Friends were contacts of cases who spent time within a closely connected network of young men who exhibited similar cannabis-using behaviour. Other contacts were family or relatives and others not closely associated with the network. Contacts were evaluated for TB including a TST	11 TB cases (8 were AFB sputum smear positive), 121 contacts (54 friends; 67 other)	8/11 TB patients were born in East Africa. Median age: 22 years	After those with a past positive TST were removed, 14 (64%) of 22 screened friends and 6 (23%) of 26 other contacts had TB infection (positive TST). The (unadjusted) risk of a positive TST result was 2.8 times greater among friends than among other contacts (95% CI:1.3–6.0). 54% friend contacts self-reported or were observed hotboxing; among those who had a TST 79% (11/14) had a positive result
Han, 2010 [19]	United States	To investigate associations between duration of use of specific illicit drugs (including cannabis) and lifetime specified health conditions (including TB)	Cross-sectional analysis of National Surveys on Drug Use and Health data for 2005–2007; a nationally representative sample of non-institutionalised civilians. Analyses were restricted to adults aged 35–49 years	Duration of cannabis use (age at initiation minus age at last use). No further details provided	Self-reported whether a doctor or other medical professional had ever diagnosed them with TB	N/A	N/A	Adults aged 35–49 years	Cannabis use (of any duration) was not associated with the risk of TB disease (reference group never used cannabis, adjusted odds ratios [aORs]: 0.79 (95% CI:0.33–1.87) for those with ≤1 year use, 0.72 (95% CI:0.25–2.06) for those with 2–10 years use, and 0.73 (95% CI:0.33–1.58) for those with ≥11 years use). Adjusted for non-medical use of prescription drugs, duration of alcohol use, duration of tobacco use, daily cigarette smoking history, age, gender, race/ethnicity, years of education, health insurance, and family income
Davis, 2017 [20]	Kazakhstan	To examine associations between various risk factors (including drug consumption) and TB	Matched case-control study. The sample comprised 562 individuals with TB (identified by the national TB programme) plus one household and one community control were identified for each index case. Study conducted June 2012 to May 2014)	Ever used cannabis; used cannabis in the past 90 days. No further details provided	Cases had to have been diagnosed with new pulmonary TB by positive culture or on clinical and radiographic grounds within 3 months before study enrolment and respond to anti-TB treatment. Household and community controls self-reported at baseline that they did not have TB	N/A	Study population consisted of 562 TB cases and 1308 controls	Participants were ≥ 18 years	In unadjusted analyses having ever used cannabis was associated with an increased odds of being a TB case (OR: 1.86, 95% CI:1.21–2.85, *p* = 0.005), as was having used cannabis in the last 90 days (OR:2.18, 95% CI:1.00–4.73, *p* = 0.049). However, in the multivariable model there was no evidence of an association with ever having used cannabis and TB (aOR:1.64, 95% CI: 0.76–3.54, *p* = 0.210) [cannabis use in last 90 days not analysed in multivariable model]. Model adjusted for: age, country of origin, marital status, education, criminal history, chronic disease, and comorbidities (HIV and diabetes mellitus)
Studies with no relevant comparator group									
Livengood, 1985 [23]	United States	To report on a community outbreak of isoniazid-resistant TB	Retrospective cohort study / outbreak investigation. Following the identification of a patient with culture and AFB sputum smear positive isoniazid-resistant TB extensive contact tracing (family, work and community contacts) was undertaken	Smoking cannabis with the index case at home, work and/or via ‘hotboxing’ in a car	Clinical and radiological diagnosis plus positive sputum smear in case	Initial close contacts plus extensive investigation of other community contacts after resistant strain realised. TST was used to identify TB in contacts (induration > 10 mm = positive TST)	One index case was identified, and a total of 104 contacts screened. One secondary case with active disease was identified	The index case was a 35-year-old male. Most of the TST-positive contacts (36/42) were associated with a local tavern frequented by the index patient	42 contacts had a positive TST result. Smoking cannabis with the index case was identified as the most important social risk factor for TB transmission - 100% (14/14) of contacts who smoked cannabis with the index case had a positive TST result
Merritt, 2007 [24]	Australia	To characterise a pulmonary TB cluster in the Hunter Area of New South Wales using a combination of traditional epidemiological methods and molecular typing	Outbreak investigation. All notifications for TB in the Hunter Area January 1994 to June 2005 were reviewed, and genotyping conducted for isolates from people who were born in Australia or New Zealand	No information	Cluster cases were confirmed if the isolate genotype was indistinguishable by MIRU, spoligotyping and IS6110 RFLP analysis. Cases missing only the RFLP analysis, or with an RFLP pattern that differed by only one band, were included if an epidemiological link was confirmed	For each case contact tracing records were reviewed and the person re-interviewed where traceable. Additional contacts were sought, and previous uncontactable contacts were followed up	9 cases were identified to be part of the cluster, and two probable cases. Number of contacts not reported	Cluster cases were people born in Australia or New Zealand. The median age at diagnosis was 35 years.8 of the 11 cases were women	Cannabis use was identified among six of the nine confirmed cases and both of the probable cluster cases
Sterling, 2000 [10]	United States	To describe a TB outbreak among a highly mobile population	Outbreak investigation. Active TB cases were identified following referral (to the Baltimore Chest Clinic), June 1998 to January 2000, and through a DNA fingerprint database for isolates matching the outbreak isolate. Contacts were identified through traditional means and additional investigations	No information	Cases were culture confirmed TB with an identical DNA fingerprint, or clinical (culture-negative) TB cases with epidemiological links to outbreak cases	Close contacts named by cases, and those identified during TB treatment visits to the home of the source case, were investigated for TB infection or disease. Work-site screenings of close contacts was conducted, and location-based screening in a nightclub frequented by source cases. TST was performed to identify TB infection	20 outbreak cases (18 culture-confirmed); and 114 contacts (77 of whom were screened for TB) were identified	Cases were predominantly young (median age 24 years), black African-American (95%), and male, 11/20 were HIV-positive	35% (7/20) of TB cases reported cannabis use
Evans, 2011 [26]	United Kingdom	To describe the identification of, and risk factors for, the single most prevalent TB strain in one area of the UK	All TB isolates in the West Midlands region (2004–2008) were genotyped to identify the most prevalent strain. Two epidemiological investigations were then undertaken. The second of these (reported on here) was an analysis of case notes of a cohort of patients in one city of the region with an indistinguishable MIRU-VNTR profile	No information	Cases were culture-confirmed and clinically diagnosed patients with the MIRU-VNTR profile of the “Mercian” strain, 2003–2006. Comparison group was culture-confirmed cases with other strains of TB diagnosed in 2004	N/A	Analyses were conducted on 35 patients with the Mercian strain, compared with 47 patients with non-Mercian strains of TB	Those with the Mercian strain of TB were more likely to be of white ethnicity and UK-born compared with those with a non-Mercian strain	In unadjusted analyses cannabis use was associated with an increased odds of having the Mercian strain: 11/35 (31%) of those with the Mercian strain reported cannabis use compared with 2/47 (4%) of those with a non-Mercian strain (OR: 10.02, 95% CI:1.96–100.33, p < 0.01). In adjusted analyses excess alcohol use and cannabis use were assessed as a combined variable. The two factors combined were associated with an increased odds of having the Mercian strain (aOR 6.26, 95% CI:1.45–27.02, p = 0.01), after adjusting for whether UK-born, previous contact with TB case, and presence of cavitary disease
McElroy, 2003 [25]	United States	To evaluate whether social network analysis can provide insights into transmission settings that might otherwise go unrecognized by routine practice	Retrospective cohort study / outbreak investigation. A cluster of 22 TB patients diagnosed between 1994 and 2001 was investigated. All adult outbreak-associated cases (*n* = 19) and selected named and unnamed contacts (*n* = 26) were re-interviewed using a network analysis questionnaire	Cannabis use in the routine investigation. Drug use practices (including ‘shotgunning’) in network analysis sample	Cases were the adult outbreak-associated TB patients included in the routine contact investigation	Primary contacts - those identified by TB cases and interviewed at time of routine investigation (1994). Secondary contacts named by either primary contacts or cases at time of network analysis interviews (2001) and those identified during original contact investigation. TST performed on contacts (induration ≥5 mm = positive TST)	19 adult TB cases were part of the cluster. 7/9 female cases had sputum acid-fast bacilli smear-positive cavitary pulmonary TB. 90 primary contacts were identified, of whom 26 were selected (convenience sample) for interview	19 adult cases included 10 men and nine women. The 7 female patients with sputum AFB smear-positive cavitary pulmonary TB worked as exotic dancers in a specific geographic area. Epidemiological links to female patients had previously been established for the majority of male patients. Median age of cases was 35 years	The convenience sample of primary contacts included 15 TST-positive and 11 TST-negative contacts. 79% of the 19 active TB cases reported cannabis use, compared with 35% of all primary contacts (33% of those TST positive and 36% of those TST negative) [authors state that “As this is not a standard case control study, the comparison of cases and primary contacts in this instance is purely descriptive”]. 44% of the 9 female cases reported ‘shotgunning’ (all used crack but it is not clear how many also used cannabis)

*TB* Tuberculosis, *TST* Tuberculin Skin Test, *OR* Odds Ratio, *aOR* Adjusted Odds Ratio, *CI* Confidence Interval, *AFB* Acid-Fast Bacilli

### Risk of bias assessments

Risk of bias was assessed for studies which utilised a comparator group. Each distinct result from these studies was assessed across six risk of bias domains using an adapted version of the ROBINS-I tool (“Risk of Bias in Non-randomised Studies – of Interventions”), [17] informed by the preliminary ROBINS-E tool (“Risk of Bias In Non-randomised Studies – of Exposure”) [18]. Although ROBINS-I is designed to assess risk of bias in intervention studies many of the domains are relevant to non-randomised studies of exposures. Summary level judgements were made for each domain and were used to inform an overall risk of bias judgement (see Additional file 2 for guidance used to make domain level risk of bias judgements).

## Results

### Description of studies

**Fig. 1:**
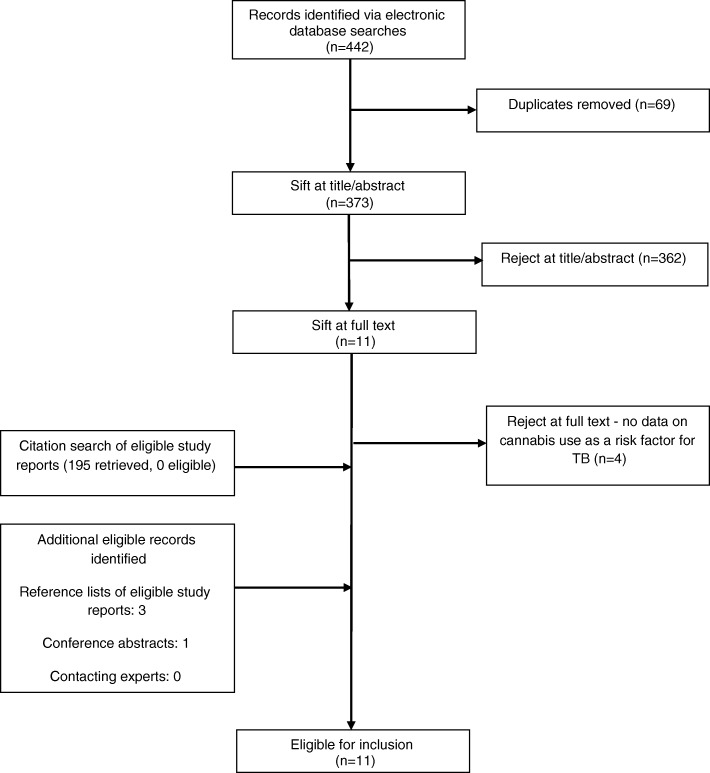
Study selection flow chart

After removal of duplicates, the initial electronic searches identified 373 potentially eligible records, with four additional eligible records identified from other sources. After screening, 11 studies fit the criteria for inclusion in the review (Fig. 1). These originated from the United States (six), Australia (three), the UK (one) and Kazakhstan (one). Study designs were heterogeneous. Six studies utilised a relevant comparator group [9, 11, 19–22]. Of these, two were retrospective cohort studies of TB outbreaks [9, 11], two used routinely collected data [19, 21], one was a matched case-control study [20], and one a case series study that included a retrospective cohort study of contacts [22]. These six studies contributed seven effect estimates, as Davis et al [20] contributed two separate results. Of the remaining five studies four were descriptive outbreak reports/investigations [10, 23–25] and one was an analytic study [26] where the outcome of interest was having TB disease from which a specific strain of TB was isolated (as compared with having TB caused by a different strain) (Table 1).

### Risk of bias in studies which utilised a relevant comparator group

Risk of bias assessments for each of the six studies which used a comparator group are presented in Table 2. All seven results (reported by the six studies) were judged to be at “Serious” risk of bias overall. These overall “Serious” judgments were largely due to the ‘Bias due to confounding’ domain, ‘Bias in selection of participants into the study’ domain, and the ‘Bias in measurement of outcomes’ domain. Two studies were at “Serious” risk of bias due to the extent of missing data.

**Table 2:** Risk of bias assessments

Author, year	Domain-level judgments						Overall judgement
	Bias due to confounding	Bias in selection of participants into the study	Bias in classification of exposures	Bias due to missing data	Bias in measurement of outcomes	Bias in selection of the reported result	
Morano, 2014 [21]	Moderate	Moderate	Moderate	Serious	Moderate	Moderate	Serious
Munckhof, 2003 [11]	Moderate	Serious	Moderate	Serious	Low	Moderate	Serious
Thu, 2014 [22]	Serious	Serious	Moderate	No information	Low	Moderate	Serious
Oeltmann, 2006 [9]	Serious	Serious	Moderate	No information	Low	Moderate	Serious
Han, 2010 [19]	Moderate	Moderate	Moderate	No information	Serious	Moderate	Serious
Davis, 2017^a^ [20]	Moderate	Low	Moderate	No information	Serious	Moderate	Serious
Davis, 2017^b^ [20]	Serious	Low	Moderate	No information	Serious	Moderate	Serious

^a^ Ever used cannabis

^b^ Cannabis use within last 90 days

### Results of studies which utilised a relevant comparator group (Table 1)

#### Cannabis use as a risk factor for latent TB

Four studies reported on the association between cannabis use and the risk of latent TB. Morano et al analysed data routinely collected by a mobile medical clinic and found cannabis use (‘ever’) to be associated with incident (but not prevalent) latent TB infection after adjustment for confounders (adjusted odds ratio [aOR]: 1.57; 95% confidence interval [CI]:1.05–2.37) [21].

One retrospective cohort study [11] and one retrospective case series combined with a retrospective cohort study of contacts [22] assessed the association between sharing a cannabis bong with a TB case and the odds of having latent TB. In the cohort study of 149 identified contacts, 45 had shared a cannabis bong with a case. The aOR for latent TB in those who shared a bong with a pulmonary TB case vs. those who had not was 2.22 (95% CI:0.96–5.17). In this cluster, cannabis smoking was frequently performed in closed rooms or cars. The authors noted that it was not possible to disaggregate relative contributions of sharing a bong and prolonged confinement in a shared airspace [11]. In the case series all three cases were young adults who reported regularly smoking cannabis through a bong. Of 111 contacts screened, 34 were positive for latent TB (positive TST), one of whom developed active TB. Contacts who shared a bong with an active TB case (*n* = 7) had a six-fold risk of a positive TST (OR 6.5, 95% CI:1.4–30.4, *p* = 0.016), though there was no adjustment for confounders [22].

One retrospective cohort study reviewed the association between being a member of a closely connected network of young men ‘friends’ who exhibited similar cannabis using behaviour [9]. There were 11 culture-confirmed cases (8 were sputum acid-fast bacilli (AFB) sputum smear positive), all of whom reported frequent ‘hotboxing’ (smoking cannabis in a confined space, such as a car, to maximise the effect [27]). The risk ratio of positive TST in the ‘friends’ contacts vs. other contacts was 2.8 (95% CI:1.3–6.0). Contacts with a past positive TST result were excluded from the analyses but no adjustment was made for confounding*.*

#### Cannabis use as a risk factor for active TB disease

Two studies reported on the association between cannabis use and having active TB disease. An analysis of routinely collected data found no evidence that cannabis use was associated with the risk of ever being diagnosed with TB disease; using those who had never used cannabis as the reference, the aORs were 0.79 (95% CI:0.33–1.87) for those with ≤1 year use, 0.72 (95% CI:0.25–2.06) for those with 2–10 years use, and 0.73 (95% CI:0.60–3.28) for those with ≥11 years use [19]. A matched case-control study also demonstrated that after adjustment for confounding factors there was no statistical evidence of an association with ever having used cannabis and recent TB disease (aOR:1.64, 95% CI:0.76–3.54, *p* = 0.210 [20].

### Results of studies which did not utilise a relevant comparator group (Table 1)

#### Cannabis use as a risk factor for latent TB

Livengood et al conducted an investigation of contacts of a patient with culture and AFB sputum smear positive isoniazid-resistant TB [23]. Cannabis use was identified as the most important social risk factor for TB transmission - 100% (14/14) of those contacts who used cannabis had a positive TST result. The authors report that the practice of ‘hotboxing’ undoubtedly contributed to TB transmission.

#### Cannabis use as a risk factor for active TB disease

Four studies reported on cannabis use as a risk factor for active TB disease. Merritt et al characterised a cluster of nine cases and two probable cases of pulmonary TB [24]. Cannabis use was identified in 67% (6/9) of confirmed cases and both the suspected cases. Authors state that use of shared smoking equipment was not explored.

Sterling et al reported on a TB outbreak among a highly mobile population in the US. The index case had AFB smear positive pulmonary disease. Twenty outbreak cases were identified, 35% of whom (*n* = 7) reported cannabis use [10].

Evans et al reviewed the association between cannabis use and having a specific TB strain by examining the epidemiological characteristics of culture-positive TB cases with an indistinguishable MIRU-VNTR profile (the “Mercian” strain) [26]. Eleven of the 35 cases (31%) with the Mercian strain reported cannabis use compared with 2/47 (4%) with other TB strains (OR: 10.02, 95% CI:1.96–100.3, *p* < 0.01).

Finally, McElroy et al investigated a cluster of 22 outbreak-associated TB patients (including 19 adults); 79% of the 19 active TB cases reported cannabis use, compared with 35% of a convenience sample of primary contacts (33% of those TST positive and 36% of those TST negative). Sputum AFB status was reported for female cases (*n* = 9) only; seven had smear-positive cavitary pulmonary TB [25].

## Discussion

We found 11 studies investigating the association between cannabis use and TB infection or disease. All studies were observational and most originated from high-income settings (10/11 studies). Six studies utilised a relevant comparator group. Four of these investigated the association between cannabis use and latent TB infection; all provided some evidence of an association, although only two of these had adjusted for confounders. The remaining two comparator studies investigated the association between cannabis use and active TB disease; neither found evidence of an association after adjusting for confounding. Five studies did not utilise a relevant comparator group; all indicated that TB outbreaks do occur among cannabis users but were not designed to test for an association.

Overall the quality of the evidence was weak and of insufficient quality to reliably quantify the risk. There was heterogeneity across populations studied and approaches to data analysis. Description of the exposure in terms of type and quantity, frequency, timing and method of cannabis use was largely inadequate. All six comparative studies were assessed as being at “Serious” risk of bias. Three of the seven results obtained from the six studies did not adjust for confounding. This may be indicative of the contexts in which studies were conducted - three emanated from outbreak investigations and the remainder utilised existing data sources not specifically designed to test this hypothesis. Where confounding variables were adjusted for these were heterogeneous across studies. Three results were assessed as being at “Serious” risk of ‘bias in the measurement of outcomes’, e.g. because investigators relied on self-reported TB status. In general, there was a lack of clear and sufficiently comprehensive information reported – e.g. for five of the seven results we could not assess ‘bias due to missing data’ as no relevant data was available. It is important that the poor quality of the available studies, with respect to addressing the review question, is borne in mind and care taken not to over interpret the data presented in this review.

The exposure and outcome measures used were not always appropriate for assessing an association between cannabis use and TB. For instance, several studies recorded the exposure as ‘ever used’ cannabis or the outcome as ‘ever’ having a diagnosis of TB. We felt it appropriate to include such studies in the review, particularly given the paucity of evidence but note that any association between cannabis and TB will likely differ depending on whether use is current or past and whether cannabis use preceded TB diagnosis. Additionally, studies lacked information on whether cannabis was used alone or with tobacco. Smoking tobacco is known to be associated with TB risk [15]. Practice varies globally - cannabis is commonly mixed with tobacco in Europe but is much more likely used alone in the US, while in Australia both practices are equally used [28]. Most studies in this review originated from the US (six), followed by Australia (three). These studies, particularly those from the US, and the two from Australia reporting on cannabis smoking via a water pipe, are therefore likely to provide some evidence, though not necessarily exclusively, relating to the specific effect of cannabis (rather than tobacco, or the combination of the two) on TB risk.

The aim of this review was to establish whether there is evidence of an association between cannabis use and TB acquisition, rather than to elicit the mechanisms by which any such association might operate. Indeed, with the evidence available it is not possible to disentangle the relative contributions of TB transmission being facilitated via close contact/shared air space (e.g. via ‘hotboxing’), the spread of TB via contaminated equipment (e.g. via shared water pipes) or cannabis use as a more general social risk factor for TB, as compared with the potential biological or physiological mechanisms through which the act of inhaling cannabis may itself increase susceptibility to TB (e.g. through damage to the lungs).

The presence of AFB in the sputum is an important risk factor for onward transmission of TB [29, 30]. None of the studies in this review directly investigated whether sputum smear status influenced the association between cannabis use and TB. However, of the six studies which used a relevant comparator group, three reported that either all or most of the active TB cases were AFB smear positive. All of these three studies reported some evidence of an association between cannabis use and TB acquisition [9, 11, 22].

### Strengths and limitations of the review process

We conducted a comprehensive literature search, with no language or date restrictions in order to ensure, as far as possible, that we identified all relevant studies. Though not a limitation of the review process as such, we note that publication bias is a potential concern – outbreak investigations are primarily purposed for disease control and many remain unpublished in any form, particularly if there are no unusual findings. Two authors independently assessed each study for eligibility, extracted the data and conducted the risk of bias assessments. This approach will have helped minimise both errors and potential biases in the review process.

ROBINS-I is a comprehensive published tool for assessing risk of bias in non-randomised studies. Since the tool is not specifically designed for assessing studies of exposures, we recognise that it may be limited in its ability to accurately classify the risk of bias in such studies. To help mitigate this we performed assessments at the domain level without answering the intra-domain signalling questions that are tailored to assessing intervention studies. We also used brief preliminary guidance for the forthcoming ROBINS-E tool tailored for studies of exposures. These approaches should reduce the potential for misclassification of bias judgements introduced by the inconsistency between study design and assessment tool. We do, however, recognise that any such risk of bias assessments may be open to subjective interpretation particularly where detailed information about study methodology is not reported, as was the case for several studies in this review.

## Conclusions

The evidence for an association between cannabis use and TB is weak. We found a lack of studies on the topic, and those that are available are of poor quality. We found some evidence for an association between cannabis use and latent TB infection, but little evidence for an association with TB disease. Many of the studies we reviewed emanated directly from field investigations of TB clusters and outbreaks including contact tracing, with the aim to identify the source and transmission routes to prevent further spread. Consequently, these were not designed specifically to assess the association between cannabis use and TB and suffered from bias at the design, conduct and analysis stages. However, a possible association between cannabis use and TB has been indicated in several outbreak investigations and it cannot be excluded as a possible risk factor based on the current literature. Indeed this remains an important question to answer to inform future outbreak control measures, especially in light of potential increased cannabis use in the context of decriminalisation policies [31].

### Implications for practice and research

The evidence base on cannabis use and TB needs to be strengthened. Opportunities exist in the context of whole-genome sequencing to more accurately distinguish cases that are part of a recent transmission cluster. The introduction of universal minimum standards for the reporting of infectious disease outbreaks could help improve the utility of published outbreak reports and must include accurate and comparable information on exposures, including, in this case, cannabis use practices, dose, frequency and timing. Efforts to facilitate observational epidemiological studies that include cannabis and other key exposures are needed and must include the collection and adjustment of key confounding factors. Studies designed to assess the independent association of cannabis use on TB infection and disease are ultimately required in order to specify the relative risk of this behaviour in the context of closely related risk factors such as close proximity and shared airspace.

## Additional files

Additional file 1: Search strategies. (DOCX 13 kb)

Additional file 2: Guidance used to make domain level risk of bias judgements. (DOCX 18 kb)

## Data Availability

The data extracted and analysed are included in this published article and additional files.

## Abbreviations

AFB: Acid-Fast Bacilli
aOR: Adjusted odds ratio
CI: Confidence interval
OR: Odds ratio
TB: Tuberculosis
TST: Tuberculin Skin Test
