# Supplementary material for: Cannabis use and the risk of tuberculosis: a systematic review
Source: BMC Public Health. 2019 Jul 27;19:1006. doi: 10.1186/s12889-019-7127-0 (PMC6660970; doi:10.1186/s12889-019-7127-0)
Supplement: Supplementary file 1 — Search strategies. (DOCX 13 kb) [file 12889_2019_7127_MOESM1_ESM.docx]

**Additional file 1. Search strategies**

**Ovid MEDLINE**

1 Cannabis/

2 Cannabis Smoking/

3 Cannabis Abuse/

4 exp Cannabinoids/

5 (cannabi* or cannabis or marihuana or hash or hashish or skunk or ganja or sinsemilia).ti,ab,kf,rn.

6 (smok* adj2 (joint* or blunt*)).ti,ab,kf.

7 (bong* or bubbler*).ti,ab,kf.

8 ((hot adj1 box*) or hotbox* or hot-box*).ti,ab,kf.

9 (drug? adj1 smok*).ti,ab,kf.

10 (pipe? adj1 (shar* or us*)).ti,ab,kf.

11 or/1-10

12 Mycobacterium tuberculosis/

13 exp Tuberculosis/

14 Tuberculosis, Multidrug-Resistant/

15 Tuberculin Test/

16 (tubercul* or TB or MTB or Koch* disease).ti,ab,kf.

17 or/12-16

18 11 and 17

19 *substance-related disorders/ and *Mycobacterium tuberculosis/

20 (tubercul* and (substance adj (use* or abuse*))).ti.

21 or/18-20

22 remove duplicates from 21

**PsycINFO**

1 exp cannabis/

2 cannabis.sh.

3 exp Cannabinoids/

4 (cannabi* or cannabis or marihuana or hash or hashish or skunk or ganja or sinsemilia).ti,ab,id.

5 (smok* adj2 (joint* or blunt*)).ti,ab,id.

6 (bong* or bubbler*).ti,ab,id.

7 ((hot adj1 box*) or hotbox* or hot-box*).ti,ab,id.

8 (drug? adj1 smok*).ti,ab,id.

9 (pipe? adj1 (shar* or us*)).ti,ab,id.

10 or/1-9

11 exp tuberculosis/

12 (tubercul* or TB or MTB or Koch* disease).ti,ab,id.

13 or/11-12

14 10 and 13

15 (tubercul* and (substance adj (use* or abuse*))).ti.

16 *"Substance Use Disorder"/ or *Drug Abuse/

17 (11 or 12) and 16

18 14 or 15 or 17

**EMBASE**

1 cannabis addiction/ or cannabis/

2 "cannabis use"/ or cannabis smoking/

3 exp cannabinoid/

4 (cannabi* or cannabis or marihuana or hash or hashish or skunk or ganja or sinsemilia).ti,ab,kw,rn.

5 (smok* adj2 (joint* or blunt*)).ti,ab,kw.

6 (bong* or bubbler*).ti,ab,kw.

7 ((hot adj1 box*) or hotbox* or hot-box*).ti,ab,kw.

8 (drug? adj1 smok*).ti,ab,kw.

9 (pipe? adj1 (shar* or us*)).ti,ab,kw.

10 or/1-9

11 exp mycobacterium tuberculosis complex/

12 exp tuberculosis/

13 (tubercul* or TB or MTB or Koch* disease).ti,ab,kw.

14 or/11-13

15 10 and 14

16 exp *"smoking and smoking related phenomena"/

17 11 and 16

18 15 or 17
