# Supplementary material for: Cannabis use and the risk of tuberculosis: a systematic review
Source: BMC Public Health. 2019 Jul 27;19:1006. doi: 10.1186/s12889-019-7127-0 (PMC6660970; doi:10.1186/s12889-019-7127-0)
Supplement: Supplementary file 2 — Guidance used to make domain level risk of bias judgements. (DOCX 18 kb) [file 12889_2019_7127_MOESM2_ESM.docx]

**Additional file 2 *-* Guidance used to make domain level risk of bias judgements**

These criteria were informed by existing guidance for the published Risk of Bias in Non-randomised Studies – of Interventions (ROBINS-I)[17] assessment tool and by provisional guidance for the forthcoming Risk of Bias in Non-randomised Studies – of Exposures (ROBINS-E) tool.

| **Bias due to confounding** | |
| --- | --- |
| **Low**  **(Unusual)** | No confounding expected. |
| **Moderate** | Confounding expected, and majority of important confounding domains have been appropriately measured and controlled for;  **AND**  Reliability and validity of measurement of important domains were sufficient, such that we do not expect serious residual confounding. |
| **Serious** | Majority of important domain have not been appropriately measured or controlled for;  **OR**  Reliability or validity of measurement of an important domain was low enough that we expect serious residual confounding. |
| **Critical** | Confounding inherently not controllable.  **OR**  The use of negative controls strongly suggests unmeasured confounding. |
| **NI** | No information on whether confounding might be present.  [**Note**: in all studies relevant to this review, confounding will almost always be present. Therefore, if study does not detail whether/how they adjusted for confounding, code it as Serious/Critical rather than NI]. |
|  | |
| **Bias in selection of participants into the study** | |
| **Low** | All participants who would have been eligible for the target experiment were included in the study;  **AND**  For each participant, start of follow up and start of exposure coincided. |
| **Moderate** | Selection into the study may have been related to exposure and outcome;  **AND**  The authors used appropriate methods to adjust for the selection bias;  **OR**  Start of follow up and start of exposure do not coincide for all participants;  **AND**  The proportion of participants for which this was the case was too low to induce important bias;  **OR**  The authors used appropriate methods to adjust for the selection bias;  **OR**  The review authors are confident that the rate (hazard) ratio for the effect of exposure remains constant over time. |
| **Serious** | Selection into the study was related (but not very strongly) to exposure and outcome;  **AND**  This could not be adjusted for in analyses;  **OR**  Start of follow up and start of intervention do not coincide (a potentially important amount of follow-up time is missing from analyses);  **AND**  The rate ratio is not constant over time. |
| **Critical** | Selection into the study was very strongly related to exposure and outcome;  **AND**  This could not be adjusted for in analyses;  **OR**  A substantial amount of follow-up time is likely to be missing from analyses;  **AND**  The rate ratio is not constant over time. |
| **NI** | No information is reported about selection of participants into the study or whether start of follow up and start of exposure coincide. |
|  | |
| **Bias in classification of exposures** | |
| **Low** | Exposure status is well defined. The exposure surrogate is well defined and comparable to that specified in the target experiment in this population. The exposure assessment methods lead to reliable classification (or discrimination) with respect to ever-exposure, exposure level, timing, or other relevant metrics.  **AND**  Exposure definition is based solely on information that reflects exposure prior to outcome assessment |
| **Moderate** | The exposure surrogate is well defined but is not comparable to that specified in the target experiment in this population.  **OR**  Exposure assessment methods lead to reliable classification or discrimination with respect to ever/never exposure, but provide no further information on exposure levels, timing, or other relevant metrics. While the metrics may be less than ideal, there is some information on exposure setting, but not in detail.  **OR**  Some aspects of the exposure status were difficult to assess. |
| **Serious** | Exposure status is not well defined;  **OR**  Major aspects of the exposure status were determined in a way that could have been affected by knowledge, presence, or risk of the outcome. |
| **Critical**  **(Unusual)** | An extremely high amount of misclassification of exposure status, e.g. because of unusually strong recall biases. Exposure assessment is not at the individual level or is not representative of individual exposure.  **OR**  The study poorly discriminates between exposed and non-exposed and among exposure categories |
| **NI** | No definition of exposure or no explanation of the source of information about exposure status is reported |
|  | |
| **Bias due to missing data** | |
| **Low** | Data were reasonably complete;  **OR**  Proportions of and reasons for missing participants were similar across exposure groups;  **OR**  The analysis addressed missing data and is likely to have removed any risk of bias. |
| **Moderate** | Proportions of and reasons for missing participants differ slightly across exposure groups;  **AND**  The analysis is unlikely to have removed the risk of bias arising from the missing data. |
| **Serious** | Proportions of missing participants differ substantially across exposures;  **OR**  Reasons for missingness differ substantially across exposures;  **AND**  (ii) The analysis is unlikely to have removed the risk of bias arising from the missing data;  **OR**  Missing data were addressed inappropriately in the analysis;  **OR**  The nature of the missing data means that the risk of bias cannot be removed through appropriate analysis. |
| **Critical**  **(Unusual)** | There were critical differences between exposures in participants with missing data;  **AND**  Missing data were not, or could not, be addressed through appropriate analysis. |
| **NI** | No information is reported about missing data or the potential for data to be missing (e.g. if the study doesn’t present a percentage for the number of identified contacts who completed screening) |
|  | |
| **Bias in measurement of outcome** | |
| **Low** | The methods of outcome assessment were comparable across exposure groups;  **AND**  The outcome measure was unlikely to be influenced by knowledge of the exposures experienced by study participants (i.e. is objective) or the outcome assessors were unaware of the exposures received by study participants;  **AND**  Any error in measuring the outcome is unrelated to exposure status. |
| **Moderate** | The methods of outcome assessment were comparable across exposure groups;  **AND**  The outcome measure is only minimally influenced by knowledge of the exposure received by study participants;  **AND**  Any error in measuring the outcome is only minimally related to exposure status. |
| **Serious** | The methods of outcome assessment were not comparable across exposure groups;  **OR**  Error in measuring the outcome was related to exposure status.  **OR**  The outcome measure was subjective (i.e. vulnerable to influence by knowledge of the exposure received by study participants);  **AND**  The outcome was assessed by assessors aware of the exposure received by study participants; |
| **Critical** | The methods of outcome assessment were so different that they cannot reasonably be compared across exposure groups. |
| **NI** | No information is reported about the methods of outcome assessment. |
|  | |
| **Bias in selection of reported results** | |
| **Low** | There is clear evidence (perhaps from examination of a pre-registered protocol or statistical analysis plan, ethics committee submission or funding application) that all reported results correspond to all intended exposures, outcomes, analyses and sub-cohorts. |
| **Moderate** | The exposure measurements and analyses are consistent with an a priori plan;  **OR**  The exposure measurements and analyses are clearly defined and both internally and externally consistent;  **AND**  The outcome measurements and analyses are consistent with an a priori plan;  **OR**  The outcome measurements and analyses are clearly defined and both internally and externally consistent;  **AND**  There is no indication of selection of the reported analysis from among multiple analyses;  **AND**  There is no indication of selection of the cohort or subgroups for analysis and reporting based on the results. |
| **Serious** | Outcomes are defined in different ways in the methods and results sections, or in different publications of the study;  **OR**  There is a high risk of selective reporting from among multiple analyses;  **OR**  The cohort or subgroup is selected from a larger study for analysis and appears to be reported based on the results. |
| **Critical** | There is evidence or strong suspicion of selective reporting of results;  **AND**  The unreported results are likely to be substantially different from the reported results. |
| **NI** | There is too little information to make a judgement (for example, if only an abstract is available for the study). |
